# COVID-19, Alcohol Consumption and Stockpiling Practises in Midlife Women: Repeat Surveys During Lockdown in Australia and the United Kingdom

**DOI:** 10.3389/fpubh.2021.642950

**Published:** 2021-06-30

**Authors:** Emma R. Miller, Ian N. Olver, Carlene J. Wilson, Belinda Lunnay, Samantha B. Meyer, Kristen Foley, Jessica A. Thomas, Barbara Toson, Paul R. Ward

**Affiliations:** ^1^College of Medicine and Public Health, Flinders University, Adelaide, SA, Australia; ^2^School of Psychology, The University of Adelaide, Adelaide, SA, Australia; ^3^School of Psychology and Public Health, La Trobe University, Melbourne, VIC, Australia; ^4^Olivia Newton-John Cancer Wellness and Research Centre, Austin Health, Melbourne, VIC, Australia; ^5^School of Public Health and Health Systems, University of Waterloo, Ontario, ON, Canada

**Keywords:** COVID-19, alcohol, midlife women, health perception, survey

## Abstract

**Introduction:** This project examined the impact of COVID-19 and associated restrictions on alcohol practises (consumption and stockpiling), and perceptions of health risk among women in midlife (those aged 45–64 years).

**Methods:** We collected online survey data from 2,437 midlife women in the United Kingdom (UK) and Australia in May 2020, recruited using a commercial panel, in the early days of mandated COVID-19 related restrictions in both countries. Participants were surveyed again (*N* = 1,377) in July 2020, at a time when COVID-19 restrictions were beginning to ease. The surveys included the Alcohol Use Disorder Identification Test—Consumption (AUDIT-C) and questions alcohol stockpiling. Analysis involved a range of univariate and multivariate techniques examining the impact of demographic variables and negative affect on consumption and acquisition outcomes.

**Results:** In both surveys (May and July), UK women scored higher than Australian women on the AUDIT-C, and residence in the UK was found to independently predict stockpiling of alcohol (RR: 1.51; 95% CI: 1.20, 1.91). Developing depression between surveys (RR: 1.53; 95% CI: 1.14, 2.04) and reporting pessimism (RR: 1.42; 95% CI: 1.11, 1.81), and fear/anxiety (RR: 1.33; 95% CI: 1.05, 1.70) at the beginning of the study period also predicted stockpiling by the end of the lockdown. Having a tertiary education was protective for alcohol stockpiling at each time point (RR: 0.69; 95% CI: 0.54, 0.87).

**Conclusions:** COVID-19 was associated with increases in risky alcohol practises that were predicted by negative emotional responses to the pandemic. Anxiety, pessimism and depression predicted stockpiling behaviour in UK and Australian women despite the many demographic and contextual differences between the two cohorts. Given our findings and the findings of others that mental health issues developed or were exacerbated during lockdown and may continue long after that time, urgent action is required to address a potential future pandemic of alcohol-related harms.

## Introduction

The Coronavirus Disease (COVID-19) pandemic has now been active for 1 year, at the time of writing having passed 63 million cases and causing at least 15 million deaths globally (1). Rapid transmission of the virus is due to the very high susceptibility of the population (2) and, although the majority of those infected will experience only mild symptoms (if any), the sheer number of global infections has resulted in a high absolute number of deaths and serious, and often prolonged complications related to infection (3)—particularly in vulnerable population groups such as the elderly and those with underlying comorbidities (4). Further, the impact of COVID-19 extends beyond physical health; the economic impacts of the pandemic have been considerable and borne disproportionately by already economically disadvantaged countries and population groups (5).

The rapid spread of COVID-19, which had involved all continents but Antarctica (6), has led to a wide range of public health responses around the world. The majority of the more effective responses have included measures that isolate and quarantine those infected and their close contacts, and restrict social interaction among the population by closing businesses, school and universities and closing national, and state and territory borders. The extent of curfews and wide-spread community lockdowns of various levels of stringency has varied in scope and date of implementation across and within countries (7). The individual and social costs of these restrictions have been the subject of concern, particularly in the area of mental health and reductions in preventive care for non-COVID-19 health conditions such as breast cancer (8–11). For instance, there was a 30% reduction in mammograms conducted through BreastScreen Australia's program between January and June in 2020 relative to the same time in the previous reporting period (12).

In ongoing investigations, using diverse methods, our team is exploring the way that midlife women, defined here as those aged 45–65 years, understand and negotiate the breast cancer risk associated with alcohol consumption. The use of alcohol is high in these women relative to other age groups, as is the incidence of breast cancer in Australia (13–16). There are many health impacts attributable to alcohol, which the World Health Organization estimates directly contributes to more than 200 health conditions including injury, mental health disorders, strokes and cardiovascular disease (17). In Australia in 2010, the social cost of alcohol (productivity, health resources, and criminal justice system combined) was estimated to be more than 14 billion Australian dollars (18)—nearly 13 USD in 2010. As is discussed by Milic et al. (19), women are more susceptible than men to the many health impacts of alcohol and also more likely to develop alcohol disorders. Alcohol has a dose-response relationship with the development of breast cancer, and has been identified as the biggest modifiable risk factor for breast cancer globally (20). Our work suggests that alcohol consumption in midlife women is mediated by both external and internal factors including socioeconomic status, work and societal role pressures, coping styles, and risk perceptions (14, 15, 21). A further significant stressor in midlife women are the psychological, emotional, physical and role transitions occurring throughout the perimenopausal period (19, 22). Within this period, menopause is associated with an array of distressing symptoms that have a substantial effect on quality life occurring at an age (global average 46–52 years) when many women remain engaged in work, are actively childrearing and have other caring responsibilities (23). The physiological changes and psychological distress associated with menopause are thought to be pivotal in the convergence of male and female alcohol consumption in midlife (19). Women have described using alcohol to assist in achieving happiness and negotiating unhappiness over the life course (14), with acute risks and stressors more strongly associated with alcohol consumption, and any changes to consumption, than the longer term potential risk of breast cancer (15).

The pandemic represents a potential modifier of alcohol behaviour and perceptions of the longer-term risk of breast cancer, particularly in the presence of a new and more immediate health risk. In our recent qualitative analyses, we describe how the risk horizons of midlife women contract from the uncertainties of the longer-term and refocus on the more pressing need to “get through” the pandemic (21). In the context of the COVID-19 lockdowns, it has been reported that women have increased their frequency of alcohol consumption in Australia, with managing stress being the most commonly reported reason (24). Australian data from May 2020, collected amidst the first COVID-19 outbreaks, indicated that a higher proportion of females than males (18% compared to 16%) increased alcohol consumption at this time (25). Similar reports of increases in alcohol consumption have been made in other Westernised counties, including those comprising the United Kingdom –UK (26). This raises the questions of whether changes in women's alcohol behaviours in response to COVID-19 lockdowns are driven by similar factors across countries; if those drivers remain the same or differ from those identified pre-COVID-19; and whether perceptions of short and long-term health risks have been influenced by COVID-19 related lockdowns.

This project aimed to examine the impact of COVID-19 on midlife women's alcohol consumption and their perceptions of health risk. We undertook two surveys in two countries known to have similar sociality (i.e., levels of acceptance and social norms) and cultural practises with respect to alcohol consumption (27, 28). The first survey was implemented at a time of uncertainty and potentially high anxiety due to rising COVID-19 case numbers in both countries. The second survey was implemented two months later, by which time some personal and social adaptation to the situation may reasonably have been expected, case numbers had reduced, and many social restrictions were beginning to lift in both countries. This is with the exception of Victoria, an Australian jurisdiction that was re-introducing a second lockdown in response to a local outbreak at the time of the second survey in July after a period of reduced restrictions (29).

COVID-19 cases appeared earlier in the UK relative to Australia, however by May 2020, associated lockdowns were implemented in both countries with their populations, excluding “frontline” workers, restricted in their movements (3, 30). The number of COVID-19 cases was very much higher in the UK than Australia, with confirmed cases reaching ~233,000 and 7,300 respectively by May 2020 (31). Despite this, at that time, both countries were rated at around 75 in the Oxford Stringency Index, which is a score derived from the existence of 18 indicators of government responses such as school closures and travel restrictions (32, 33). By July 2020, restrictions were just beginning to be lifted in both countries, with pubs and restaurants starting to open and with fewer mobility restrictions, although legal requirements for social distancing and associated travel limitations remained. At this time, the Oxford Stringency Index was ~65 in Australia and in the UK (33).

Data from our surveys therefore provide insight into critical points of interest: how Australian and British women's alcohol consumption changed over time during COVID-19 in relation to their perceptions of health risks.

## Materials and Methods

We conducted online surveys with 1,218 midlife women in Australia and 1,219 United Kingdom (UK) in May 2020. Participants were surveyed again (799 in Australia and 578 in the UK) in July 2020. The study was approved by the Flinders University Human Research Ethics Committee.

### Participants

The participants were recruited via a commercial panel provider, Qualtrics. The company used a quota system to recruit women aged 45–64 years with evenly distributed tertiles of household income based on ABS definitions of “low,” “medium,” and “high” (34) as determined by the most recent Census data for each country. Women with existing chronic conditions were ineligible for participation in the survey. Women identifying in this group were excluded on their response to the question “Do you currently suffer from any chronic illnesses?” and the advice that a chronic condition is “…a human health condition or disease that is persistent or otherwise long-lasting in its effects or a disease that comes with time. E.g., Diabetes, Heart Disease, Arthritis.” This group was excluded due to the potential impact that ongoing chronic health issues might have on drinking behaviours, engagement with the workforce and household and personal income. After two months, the same participants were invited to participate in a second survey. We estimated that we would need to recruit 1,200 women in each country (i.e., 2,400 participants in total) assuming that proportional estimates were approximately normally distributed and based on a precision of 4% with confidence intervals of 95%, and on the basis of an anticipated 50% attrition at follow-up (on advice from Qualtrics). Participants were provided with a non-monetary reimbursement in the form of loyalty points or vouchers (depending on the sample source) at survey completion. The reimbursement was approximately equivalent to Australian minimum wage pro-rata to survey length (~15 min).

### Surveys

In May 2020, participants in Australia and the UK completed the first online survey. The survey landing page described the study in full, explaining that individuals would be invited to respond to two surveys. The landing page also contained contact details of the research team and, consistent with the Australian National Statement on Ethical Conduct of Human Research (35), participants acknowledged having read the information and indicated their consent before proceeding with the survey. We collected a range of demographic and living arrangement information: age, relationship status, parenting status and number of children living at home, respondent education level, household and personal income, and post-code. Participants provided information on their usual employment status and whether their work status or conditions had changed because of social restrictions imposed as part of the public health response to COVID-19.

Pattern of alcohol consumption was measured using the Alcohol Use Disorder Identification Test-Consumption (AUDIT-C), which provides a total score out of 12 across three categories of drinking frequency and quantity and has been validated for use in a range of general populations (36, 37). In addition, participants were asked if alcohol patterns had changed during the pandemic and, if so, in what way (e.g., more frequently, more volume, to pass time, and other options). Information about online alcohol purchasing and context of drinking (alone or in company) was also collected. Participants were asked about taking measures to ensure access to alcohol such as buying more than usual, here defined as “stockpiling.”

As well as general health status (Overall, how would you rate your general health?—very good/good/moderate or fair), participants were asked about their status with regard to COVID-19 infection (ever diagnosed or suspected—yes/no), and history of breast cancer diagnoses (ever diagnosed—yes/no), and their self-rated likelihood that they might be diagnosed with either of these conditions in future (5-point Likert—very unlikely to very likely). To explore emotional and psychological responses to the COVID-19 pandemic, participants were provided with a list of options [fearful/anxious, depressed, more connected with people (e.g., via social media or with neighbours/local community), isolated/lonely, hopeful about the future, a reduced sense of control, pessimism about the future, and uncertainty], and asked to select any they had experienced during the COVID-19 pandemic (Have you felt any of the following during the COVID-19 pandemic?).

In July 2020, the second survey was completed by Australian and UK participants of the first survey. The second survey was shorter than the first but revisited many of the items covered in the first survey, including all of those relevant to this analysis. These included any COVID-related changes to living arrangements, work status or conditions, and patterns of alcohol purchasing and consumption. As with the first, the second survey also included the items related to breast cancer and COVID-19 status, and emotional and psychological responses to the ongoing pandemic and its restrictions.

### Data Analysis

The current analysis focused on the drivers of alcohol consumption; analyses related to social class and financial status will be the subject of further reports. Specifically, our main dependent variables were the AUDIT-C, and alcohol stockpiling behaviour [“During the COVID-19 pandemic, have you taken any measures to ensure access to alcohol (e.g., ordered alcohol online, bought more than usual)?”]. Independent variables were: loss of paid work; health risk perceptions (likelihoods of contracting COVID-19 and developing breast cancer); emotional responses to COVID-19 (fear or anxiety, depression, improved social connexion, loneliness, less in control, pessimism, and uncertainty); and subjective self-report of increased alcohol consumption since COVID-19 (“Would you say you have consumed overall more or less alcohol during the COVID-19 pandemic?”—consumed more/consumed less/consumed the same).

Data were analysed using Stata (release 15, Stata Corporation, College Station, TX, United States). Survey sample characteristics were first analysed descriptively and bivariately to assess differences between Australian and UK participants. To assess patterns that might be reflective of bias introduced by participant attrition, a comparison of all responses among those completing only the first or both surveys was undertaken and confirmed no difference in response patterns once all demographic and alcohol consumption patterns were examined. Using alcohol consumption indicators as the dependent variable, bivariate analyses consisted of Chi-square, Mann-Whitney and *t*-tests as appropriate. Relative risks and risk difference were calculated, and 95% confidence intervals constructed. Collinearity was assessed using Chi-square, Phi statistics and variance inflation factor where appropriate. McNemar's tests were also used to determine differences in proportions of outcome variables between surveys. Finally, multivariate binary logistic regressions were undertaken to determine independent predictors of alcohol behaviour. All data were analysed at the 0.05 significance level.

## Results

In the first survey, there were 1,218 Australian and 1,219 UK participants (*N* = 2437). The characteristics of the participants are presented in Table 1. Although quotas were used to ensure participants fell into equivalent tertiles of income, there were several differences in other demographic characteristics between cohorts. Notably, Australian participants were more likely to be parenting without a partner and to be tertiary educated. UK participants were more likely to report having been “usually engaged in paid work” (pre-COVID), including in full time work, and were more likely to report having “experienced changes in their work conditions as a result of COVID-19,” although these changes were most commonly due to requirements to work at home. Loss of paid work hours was more commonly reported by Australian participants at the time of the first survey. Although perceptions of the long-term risk posed by breast cancer did not differ between cohorts, women in the UK reported significantly higher perceived likelihood of contracting COVID-19.

**Table 1:** Characteristics of Australian and UK participants at entry (*N* = 2,437).

**Characteristic**	**Australia**	**UK**	^*^p-value
Age in years—median (range):	54 (45–64)	53 (45–64)	**0.020**
Completed tertiary education—*n* (%):	711 (58.4)	635 (52.1)	**0.002**
Children living at home—*n* (%):	494 (40.6)	540 (44.3)	0.062
^**^Parenting without partner—*n* (%):	333 (46.1)	241 (34.1)	** <0.001**
Number—median (range):	2 (1–10)	2 (1–10)	0.896
^†^Health risk perceptions—*n* (%)			
Likely to get COVID-19:	104 (8.7)	259 (22.7)	** <0.001**
Likely to develop breast cancer:	82 (9.2)	105 (11.0)	0.197
Usual employment status— *n*(%)			
Any paid work:	789 (64.8)	910 (74.7)	** <0.001**
Full time work:	409 (33.6)	587 (48.2)	** <0.001**
^††^Change in work conditions— *n* (%)			
Required to work from home:	133 (15.9)	239 (26.6)	** <0.001**
Lost a job:	71 (17.5)	90 (15.3)	0.359
Lost hours:	196 (24.8)	154 (16.9)	** <0.001**
Forced to take leave:	55 (7.0)	32 (3.5)	**0.001**
^‡^AUDIT-C scores—median (range):	3 (1–11)	4 (1–12)	** <0.001**
“Stockpiling” of alcohol at home*n* (%)	185 (17.9)	339 (30.4)	** <0.001**
Changes in alcohol consumption—*n* (%)			**0.001**
More likely to drink alone	316 (30.6)	273 (24.4)	
Change in physical environment from usual drinking	298 (28.8)	492 (44.1)	** <0.001**
Consumes more	246 (23.8)	361 (32.3)	
Consumes less	242 (23.4)	278 (24.9)	
Consumption unchanged	547 (52.9)	578 (42.8)	** <0.001**
^‡‡^Pattern of increased consumption since COVID-19—*n* (%)			
More frequent but same amount:	140 (56.9)	197 (54.6)	
More frequent and more alcohol:	76 (30.9)	139 (38.5)	
Same frequency but more alcohol:	30 (12.2)	25 (6.9)	**0.030**

* *Statistical tests: Mann-Whitney, Chi-square as appropriate (significance <0.05, in bold font)*.

** *Among those with children living at home (N = 1,034)*.

† *Among those not previously diagnosed with either COVID-19 (N = 2,342) or breast cancer (N = 1,846)*.

†† *Among those reporting change in work conditions since COVID-19 (N = 994)*.

‡ *Alcohol use disorder identification test—consumption, among those reporting alcohol consumption (N = 1,699)*.

‡‡ *Among those reporting increased alcohol consumption (N = 607)*.

UK participants scored higher on the AUDIT-C, with a median score of 4 indicating potentially problematic drinking frequency and were more likely to report having increased their alcohol consumption as time since COVID-19 passed relative to Australian participants. Among those reporting increased alcohol consumption, the change mainly involved greater frequency of drinking in both cohorts. Relative to Australians, UK women were also more likely to report “stockpiling” of alcohol at home as a response to the COVID-19 crisis.

The relationships between selected impacts of COVID-19 that were reported in the first survey and problematic drinking (AUDIT-C score ≥4 and stockpiling) are presented in Table 2. For both cohorts, the strongest associations were between stockpiling alcohol, consuming more alcohol and drinking at problematic levels. Drinking more alcohol during COVID-19 was associated with nearly five times the likelihood of problematic drinking in Australian women and three times the likelihood in UK women. Stockpiling of alcohol was associated with three times the likelihood of problematic consumption in Australian women, with UK women approaching a similar level of risk.

**Table 2:** COVID-19 impacts at entry, problematic drinking, and alcohol stockpiling (*N* = 2,437).

**Reported impact**	^*^AUDIT-C score ≥4—^†^RR (CI)	**Alcohol stockpiling—^†^RR (CI)**
^‡^Lost paid work since COVID-19		
Australia:	0.96 (0.80, 1.15)	1.26 (0.99, 1.60)
UK:	**1.17 (1.03, 1.32)**	**1.30 (1.03, 1.65)**
^§^Health risk perceptions		
Likely to contract COVID-19		
Australia:	1.02 (0.80, 1.31)	**1.99 (1.30, 3.03)**
UK:	1.07 (0.95, 1.21)	1.24 (0.99, 1.56)
Likely to develop breast cancer		
Australia:	**1.29 (1.03, 1.61)**	1.44 (0.88, 2.34)
UK:	1.17 (0.99, 1.37)	**1.62 1.10, 2.37)**
Responses to COVID-19		
•Fearful or anxious		
Australia:	1.05 (0.91, 1.21)	**1.96 (1.50, 2.54)**
UK:	1.05 (0.95, 1.16)	**1.47 (1.23, 1.76)**
•Depressed		
Australia:	**1.26 (1.09, 1.47)**	**2.03 (1.57, 2.63)**
UK:	**1.19 (1.07, 1.33)**	**1.58 (1.32, 1.89)**
•More socially connected		
Australia:	**1.18 (1.01, 1.40)**	1.26 (0.92, 1.72)
UK:	0.94 (0.82, 1.06)	1.20 (0.99, 1.47)
•More lonely and isolated		
Australia:	**1.26 (1.09, 1.45)**	**1.80 (1.39, 2.34)**
UK:	0.98 (0.86, 1.11)	**1.28 (1.05, 1.56)**
•Less in control		
Australia:	1.07 (0.92, 1.23)	**1.59 (1.23, 2.07)**
UK:	1.08 (0.98, 1.20)	**1.57 (1.32, 1.88)**
•Pessimistic about the future		
Australia:	1.16 (0.99, 1.36)	**1.86 (1.43, 2.41)**
UK:	1.08 (0.97, 1.20)	**1.67 (1.40, 1.98**)
^*^AUDIT-C score ≥4		
Australia:	–	**3.81 (2.80, 5.18)**
UK:	–	**3.38 (2.63, 4.35)**
Drinking more alcohol since COVID-19		
Australia:	**4.71 (3.58, 6.21)**	**3.36 (2.79, 4.06)**
UK:	**3.04 (2.53, 3.65)**	**2.79 (2.37, 3.29)**

* *Alcohol use disorder identification test—consumption*.

† *Risk Ratio (95% confidence interval)—p < 0.05 in bold font*.

‡ *Among those reporting having lost any paid work (N = 1,699)*.

§ *Among those not previously diagnosed with either COVID-19 (N = 2,342) or breast cancer (N = 1,846)*.

There were variations between cohorts and effects according to the dependent variable analysed. Higher AUDIT-C scores were associated with loss of paid work in Australian participants as was the perception of risk for breast cancer, neither of which were associated with AUDIT-C scores in UK participants. Increased perceived risk for COVID-19 was not associated with problematic drinking in either group, nor were the majority of emotional/psychological responses for which data were collected. Reported feelings of depression was associated with increased risk for problematic drinking in both participant groups. In Australian women only, higher AUDIT-C scores were associated with feeling more socially connected and, conversely, with feeling isolated and lonely.

In both cohorts, strong univariate relationships were found between most of the independent variables and stockpiling of alcohol, with the exception of feeling “more socially connected.” UK women who stockpiled were more likely to report stockpiling if they had lost paid work since the beginning of COVID-19 and also more likely to report stronger perceptions of COVID-19 risk. Australian participants who stockpiled were more likely to report susceptibility to breast cancer than those from the UK. In the first survey, participants who stockpiled from both nations were more likely to report feeling fearful or anxious, depressed, lonely and isolated, less in control, pessimistic about the future and uncertain.

### Second Survey

A total of 1,377 of the originally surveyed women participated in the second survey; 799 Australian and 578 UK women. Comparison of all demographic data collected in both surveys showed no statistical differences between samples (for both cohorts in both time periods) and therefore supported the recruitment strategy. Across surveys, the median AUDIT-C score remain the same at 4 (IQ range 2–5) for UK women and 3 (IQ range 2–5) for Australian women. Exact McNemar's tests determined that there were no statistically significant differences in the proportions of participants scoring in the problematic drinking range (≥4) between surveys in either cohort. Approximately 30% of participant scores decreased and 30% increased (around 40% were scored the same) between the two surveys, with no differences in these proportions between the two cohorts. An additional 8% of both groups reported commencing stockpiling since completing the first survey, whereas 12% reported having stopped stockpiling since completing the first survey. Although the proportion reporting stockpiling did not change between surveys in the UK, an exact McNemar's test suggested there was a significant increase in stockpiling by Australian women (*p* = 0.019).

Women reported 41 new COVID-19 infections occurring since the previous survey; 20 (2.5%) in Australian and 21 (3.9%) in UK participants. Ten of the 20 Australian cases were reported from Victoria, which was experiencing an outbreak during the time of the second survey. The incidence figures were not significantly different despite the larger case numbers and transmission risk in the UK. Perhaps due to this context, women in the UK were significantly more likely to report feeling at risk for COVID-19 than Australian participants (RR = 2.03, 95% CI: 1.54, 2.73, *p* < 0.001) although they were also more likely to report susceptibility for breast cancer (RR= 1.72, 95% CI: 1.23, 2.42, *p* = 0.002). Unlike the first survey, neither perceptions of COVID-19 or breast cancer risk were associated with AUDIT-C score or stockpiling of alcohol in either cohort. A small proportion of women reported increased fear of contracting COVID-19 between surveys (~6% in both cohorts), but this was also not associated with alcohol consumption or stockpiling. UK women were more likely than Australian women to have lost work between the two surveys (RR= 1.49, 95% CI: 1.34, 1.96, *p* = 0.004) but this was not associated with alcohol consumption (per AUDIT-C) or stockpiling of alcohol.

The reported impacts of COVID-19 identified in the second survey are presented in Table 3. In UK women, depression was associated with problematic drinking as were feelings of loneliness and isolation in Australian women. As with survey one, most negative emotional responses to the pandemic were associated more strongly with alcohol stockpiling than AUDIT-C score in both groups. Among women reporting emotional responses for the first time in survey 2, only newly reported depression was associated with problematic drinking in UK women, and with stockpiling of alcohol in both groups. As with the first survey, self-report of an increase in alcohol consumption since COVID-19 was strongly associated with both AUDIT-C score and stockpiling, particularly in Australian women, with more than seven times the risk for stockpiling.

**Table 3:** Selected COVID-19 impacts in the second survey, problematic drinking, and alcohol stockpiling (*N* = 1,377).

**Reported impact**	^*^AUDIT-C score ≥4—^†^RR (CI)	**Alcohol stockpiling—^†^RR (CI)**
•Fearful or anxious		
Australia:	1.12 (0.93, 1.35)	1.27 (0.93, 1.74)
UK:	1.09 (0.93, 1.27)	**1.53 (1.14, 2.06)**
•Depressed		
Australia:	1.11 (0.91, 1.37)	**1.72 (1.12, 2.68)**
UK:	**1.32 (1.13, 1.54)**	**1.51 (1.02, 2.24)**
•More lonely and isolated		
Australia:	**1.32 (1.09, 1.59)**	**2.04 (1.43, 2.91)**
UK:	0.98 (0.86, 1.11)	1.07 (0.75, 1.52)
•Less in control		
Australia:	1.10 (0.92, 1.33)	^‡^**1.59 (1.11, 2.27)**
UK:	1.03 (0.88, 1.21)	**1.41 (1.04, 1.91)**
•Pessimistic about the future		
Australia:	1.15 (0.95, 1.39)	**2.14 (1.50, 3.05)**
UK:	0.91 (0.77, 1.08	**1.62 (1.20, 2.19)**
•Uncertainty		
Australia:	0.93 (0.77, 1.13)	1.27 (0.86, 1.90)
UK:	1.05 (0.89, 1.23)	**1.58 (1.11, 2.5)**
Started feeling depressed since the first survey:		
Australia:	0.97 (0.73, 1.30)	**1.72 (1.12, 2.68)**
UK:	**1.39 (0.17, 1.66)**	**1.51 (1.02, 2.24)**
Drinking more alcohol since COVID-19		
Australia:	**2.33 (2.00, 2.74)**	**7.16 (4.97, 10.33)**
UK:	**1.58 (1.37, 1.82)**	**2.72 (2.02, 3.65)**

* *Alcohol use disorder identification test**—**consumption*.

† *Risk ratio (95% confidence interval)**—**p < 0.05 in bold font*.

‡ *No longer significant once Victoria (in which the residents were experiencing a second lockdown) was excluded*.

### Multivariate Analyses

AUDIT-C scores and alcohol stockpiling were strongly associated with each other; however, the predictors of the outcomes included here were more consistently linked to stockpiling. For this reason, we fit separate multivariate log binomial models to assess independent predictors of alcohol stockpiling in participants of the both surveys (Table 4). At the first time point, feeling fearful or anxious, lonely or isolated and uncertainty were no longer significantly associated with alcohol stockpiling once we had adjusted for the other emotional responses to COVID-19, specifically feelings of depression, loss of control, and pessimism about the future. Being below the median age of 54 years and being a resident of the UK also independently predicted alcohol stockpiling. Tertiary education, regardless of country, was protective against stockpiling behaviour.

**Table 4:** ^*^Independent predictors of alcohol stockpiling in midlife women in Australia and the United Kingdom—May and July 2020.

**Model 1—survey 1 (*n* = 2152)**	**Relative Risk (95% CI)**	**Risk Difference (95% CI)**	***p*-value**
Depressed	1.39 (1.19, 1.62)	0.10 (0.06, 0.15)	<0.001
Less in control	1.33 (1.14, 1.55)	0.06 (0.02, 0.10)	<0.001
Pessimistic about the future	1.43 (1.23, 1.67)	0.10 (0.05, 0.14)	<0.001
Tertiary educated	0.82 (0.71, 0.95)	−0.05 (−0.08, −0.01)	0.008
Resident of United Kingdom	1.58 (1.35, 1.83)	0.10 (0.07, 0.13)	<0.001
**Model 2—survey 2 (*****n*** **=** **1222)**	**Relative Risk (95% CI)**	**Risk Difference (95% CI)**	***p*****-value**
Fearful or anxious	1.49 (1.17, 1.89)	0.07 (0.03, 0.11)	0.001
Pessimistic about the future	1.64 (1.29, 2.07)	0.10 (0.05, 0.16)	<0.001
Tertiary educated	0.71 (0.56, 0.89)	−0.07 (−0.11, −0.02)	0.003
Resident of United Kingdom	1.53 (1.21, 1.93)	0.08 (0.03, 0.12)	<0.001

* *Both models are adjusted for age*.

In the second model (Table 4), once we adjusted for the other emotional responses to COVID-19, feeling less control, depressed, lonely or isolated, and uncertain did not retain significance in the multivariate binary regression. By survey two, stockpiling was independently predicted by feeling fearful or anxious and feeling pessimistic about the future. As with the first time point, being a UK resident also predicted alcohol stockpiling and tertiary education was protective against this behaviour.

We regressed changes in emotional responses for all variables between surveys, but only changes in depression status significantly predicted alcohol stockpiling by survey two. The age-adjusted RR for newly reported depression was 1.59 (95% CI: 1.18, 2.14, *p* = 0.002), but further modelling indicated that none of the reported changes in emotional status between surveys predicted AUDIT-C score at the second time point. Our final model investigating independent predictors across both time points for the outcome of alcohol stockpiling at the second time point is presented in Table 5. Reporting depression for the first time between surveys was strongest predictor of stockpiling in the second survey, followed by reporting pessimism and fearfulness/anxiety in the first survey. Residence in the UK predicted stockpiling at time point two and tertiary education continued to be protective in this model.

**Table 5:** ^*^Independent predictors of alcohol stockpiling at survey 2 in midlife women in Australia and the United Kingdom—May–July 2020 (*n* = 1,222).

**Predictors**	**Relative risk (95% CI)**	**Risk difference (95% CI)**	***p*-value**
Started feeling depressed between surveys	1.53 (1.14, 2.04)	0.10 (0.03, 0.18)	0.004
Pessimistic at survey 1	1.42 (1.11, 1.81)	0.08 (0.03, 0.13)	0.005
Fearful or anxious at survey 1	1.33 (1.05, 1.70)	0.05 (0.01, 0.09)	0.020
Tertiary educated	0.69 (0.54, 0.87)	−0.06 (−0.12, −0.02)	0.002
Resident of United Kingdom	1.51 (1.20, 1.91)	0.08 (0.04, 0.12)	<0.001

* *Model is adjusted for age*.

## Discussion

This study investigated the impact of COVID-19 on midlife women's alcohol consumption and perceptions of health risk in two Westernised countries with a similar sociality and culture with respect to patterns of alcohol consumption (27, 28). Our findings indicate that COVID-19 lead to more risky practises with respect to alcohol and that this was predicted by negative emotional responses to the pandemic.

The numbers of cases and rates of community transmission were very much higher in the UK relative to Australia at the time of both surveys (31, 38). It is therefore not surprising that UK participants reported heightened perceptions of personal risk with regard to COVID-19 infection. In contrast, the longer-term potential risk posed by breast cancer was similar between cohorts, notwithstanding the cancer risk associated with the more frequent alcohol consumption reported by UK women. Neither perceptions of short- or long-term health risks predicted greater alcohol consumption in either group on multivariate analyses. The impact of the lockdown itself may have been more influential than the fear of the short-term risk of contracting COVID-19, with longer term risk for breast cancer also not uppermost in mind. As discussed by Bavli et al. (39), lockdowns have been useful for limiting transmission of COVID-19, but inevitably come with a fair degree of “collateral damage” such as harms associated with delays in health investigations and treatment. As previously noted, this includes reductions in preventive care for health conditions such as breast cancer (12).

Recent data from other studies indicate that women in both countries are more likely to report increased alcohol consumption than reduced consumption since COVID-19 (25, 26). Our results extend this to indicate that residence in the UK independently predicted alcohol stockpiling, which was closely associated with alcohol consumption in our study. This relationship persisted at each time point and across the study period even after controlling for the protective effect of tertiary education, which a smaller proportion of UK respondents had completed. In discussing increased convergence in drinking between men and women in the UK, Nicholls (40) discusses the demise of the working man's pub and the rise of the “night time economy,” where all forms of alcohol consumption (pre-drinking and in pubs and clubs) comes to play an important role in “doing” gender (whether pre-drinking with friends, and in bars, pubs and clubs). Further investigation might uncover whether this may help to explain the persistence of greater alcohol consumption in UK women during lock down, where alcohol would be consumed less publicly.

Although stockpiling alcohol and problematic drinking were strongly correlated in both cohorts, the individual drivers of these behaviours were not necessarily the same. Assuming that stockpiling is an indication of “intention” to consume alcohol in the future, emotional responses to the pandemic (including depression, fear and anxiety, and pessimism) were strongly associated individually with alcohol stockpiling, but these same emotions were not necessarily associated with consumption at problematic levels, as indicated by the AUDIT-C.

The *intention* to act in the future is indicated through the purchasing of specific items, with stockpiling suggesting purchase that exceeds current use, and fear of scarcity regarding future availability of alcohol. The phenomenon of stockpiling has been reported in other research into infectious disease outbreaks (41). Moreover, the stockpiling of other items including guns, toilet paper and gold, has also been associated with higher levels of COVID-related anxiety (42). This potentially indicates that the stockpiling behaviour could be a preparation for a worsening of the pandemic and that alcohol offered the participants a chance to prepare for a worsening of the situation driven by their feelings of depression, fear and anxiety and pessimism. While this would require further research, across our study period, pessimism was a key emotional response predicting stockpiling at both time points. Our findings suggest that pessimism and anxiety at the first time point may have gradually given rise to depression. It is possible that the tedium of the pandemic and associated lockdowns ultimately “wore people down” overtime while underscoring their need to prepare for the “long haul.” As is argued by Ogden (43), people under stress may experience a distortion of time, with reduced socialisation associated with an apparent slowing of the passage of time. Robb et al. (44) found that women in the UK reported worsening depression and anxiety symptoms after lockdown, which were more severe than those reported by men. It is noteworthy that 12% of our participants reported feeling depressed and 29% reported feeling anxious or fearful at both time points. Whether these are responsive to the circumstances or represent pre-existing mental health conditions, such feelings persisting throughout the intervening period may predict persisting psychological morbidity related to the lockdown (45).

Regardless of measurable changes in AUDIT-C scores, participant perceptions of increased drinking were strongly linked to both stockpiling and problematic drinking and this was particularly evident in the Australian sample. Although a greater proportion of UK women reported that they were consuming more alcohol, the impact of COVID-19 on AUDIT-C and stockpiling was greater for Australian women with previously problematic drinking. Some of this may be due to increased availability of alcohol in Australia, even during lockdown. In recent years, the density of alcohol outlets (offering on- and off premises consumption) in Australia has increased without reference to the number of other outlets whilst the UK has been limiting alcohol licences on the basis of the local density of other alcohol selling premises (46). Although an alcoholic drink was reported to be <18 min away in both countries pre-COVID (among the shortest times in the world), bulk shopping was available from large alcohol-specific warehouses only in Australia (47). On premises alcohol consumption was not possible during lockdown, but businesses specialising in alcohol sales were considered “essential services” in both countries, with home delivery services also only available in Australia before and during COVID-19 (48, 49). Substance use issues in vulnerable populations have been noted to worsen as a direct impact of social-distancing measures (39), the significance of which is heightened by reduced access to support services that might normally be available for alcohol issues due to the lockdown (50).

Tertiary education was protective for both problematic alcohol consumption and alcohol stockpiling in both cohorts and across time. It has long been noted that education is strongly linked with improvements in nearly all health and mortality outcomes, which is thought to be attributable to higher incomes, better nutrition, less crowded housing, and increased access to health care services (51, 52). While the relationship between environmental circumstances and alcohol consumption remains equivocal, Lui et al. (53) found that education and alcohol consumption were positively correlated with each other, stating (page 4) that a “…positive SES gradient was found such that with each level of higher education, more alcohol was consumed in the past year for both genders.” More consistent with our results, however, Lui et al. (53) also found that problematic drinking such as “heavy episodic drinking” was inversely related to education level in mid-aged people (53).

We used quota sampling to recruit midlife women with similar distributions of household income, however the two populations differed significantly on most other demographic variables. Prior to COVID-19, UK women were reported to drink more alcohol per capita than Australian women (17), which aligned with our findings. Despite these differences, the emotional responses to COVID-19 that independently predicted stockpiling behaviour were strikingly similar. Globally, increased prevalences of depression and anxiety in association with COVID-19 related lockdowns have been identified (11, 39). Despite the clear increased need, the lockdowns have affected access to mental, many of which have closed with acute health services prioritising treating COVID-19 cases, particularly in countries with high infection rates (54). This situation has led to warnings that mental health could be the “next pandemic” (55), which our findings suggest could be swiftly followed by a pandemic of alcohol-related harms.

### Limitations

We used quota sampling on tertiles of income and restricted the survey to healthy women aged 45–64 years, however there were many other differences between the two cohorts. Although many of the differences of which we are aware are unlikely to have directly impacted on alcohol consumption behaviour, there are potentially a range of socio-cultural, commercial and policy related factors (for which we did not collect data) that are likely to have had direct impacts on behaviour. Our multivariate analyses informed our conclusion that “country of residence” was an independent risk factor for alcohol stockpiling, but future investigation is required to unpack the relative influence of some of the commercial and structural components of this relationship.

Perhaps unsurprisingly, we were not able to collect baseline (pre-COVID) data with which to compare changes at each time point. Although self-reported information on changes in behaviour since before COVID-19 was collected, it is important to acknowledge that our findings are most relevant to the period between the two survey points. Although the Oxford Stringency Index was similar for each country at both periods, there was substantial local variability within nations. For example, the second survey was administered at a time when a second lockdown was occurring in one jurisdiction of Australia, representing 28% of our Australian cohort at that time point. Separate analyses excluding the Victorian participants did not demonstrate any differences to our findings, however it is not possible to rule out potential influence of other variability in local and national contexts.

The survey included the well-validated AUDIT-C instrument (36, 37) to collect information about the volume and frequency of alcohol consumption and asked for subjective self-reports of increased alcohol consumption since COVID-19. As is common to many surveys, our reliance on self-reports may have led to an under-estimation of alcohol consumption. The complete anonymity of the online survey and strong likelihood that participants would completed it in private, however, may have reduced the likelihood of socially desirable responding.

Finally, subjective emotional responses to the pandemic were collected in the survey and the analyses focused on the reported feelings of depression and anxiety and fearfulness. Measuring depression and anxiety using validated psychological instruments in this survey was beyond the scope of this study, associations found between these reported feelings and alcohol behaviours should be interpreted cautiously. Nonetheless, our findings do implicate between negative emotional affect in alcohol practises and strongly suggest this as an area for future investigation.

## Conclusions

In this study, COVID-19 was associated with increases in risky alcohol practises, specifically alcohol stockpiling and problematic drinking, and this was predicted by negative emotional responses to the pandemic. Our findings suggest that pessimism and anxiety that were evident at the first time point may have gradually given rise to depression, which if persisting over time may predict more entrenched psychological morbidity. COVID-19 was associated with greater risk with respect to alcohol consumption among the already vulnerable subgroups in Australia. It is important that access to mental health support services during lockdown and beyond is enhanced. Future public health research could include how the local and national context of alcohol consumption and the actions of commercial players interact with individual decisions to stockpile as well as confirming and investigating why tertiary education seems to be protective against stockpiling.

Anxiety, pessimism and depression were emotional responses to COVID-19 that predicted stockpiling behaviour in UK and Australian women despite the many demographic and contextual differences between these two cohorts. Increasing prevalence of depression and anxiety in association with COVID-19 related lockdowns has been noted around the world, and there is growing evidence that the mental health issues developed or exacerbated during lockdown may continue long after lockdown is lifted. If mental health harms become the “next pandemic,” our findings suggest that this could be swiftly followed by a pandemic of alcohol-related harms.

## Data Availability Statement

The raw data supporting the conclusions of this article will be made available by the authors on reasonable request, without undue reservation.

## Ethics Statement

The studies involving human participants were reviewed and approved by Flinders University Human Research Ethics Committee. Written informed consent for participation was not required for this study in accordance with the national legislation and the institutional requirements.

## Author Contributions

EM contributed to the design and implementation of the study, analysed the data, and drafted the manuscript. IO, CW, BL, SM, KF, JT, and PW contributed to the study design and drafting of the manuscript. BT contributed statistical expertise to the analysis and drafting of the manuscript. All authors reviewed the manuscript prior to submission.

## Conflict of Interest

The authors declare that the research was conducted in the absence of any commercial or financial relationships that could be construed as a potential conflict of interest.
